# Analgesic Effect of Ultrasound-Guided Anterior Quadratus Lumborum Block at the L2 Level in Patients Undergoing Laparoscopic Partial Nephrectomy: A Single-Center, Randomized Controlled Trial

**DOI:** 10.1155/2022/8958859

**Published:** 2022-12-15

**Authors:** Ying He, Mingying Huang, Qiangui Zhong, Huijuan Ni, Zenggui Yu, Xinjian Zhang

**Affiliations:** ^1^Department of Anesthesiology, The Third Affiliated Hospital of Guangzhou University of Chinese Medicine, Guangzhou, China; ^2^Department of Anesthesiology, The Third Clinical Medical College of Guangzhou University of Chinese Medicine, Guangzhou, China; ^3^Department of Anesthesiology, Fujian Provincial Medical College, Fujian Medical University, Fuzhou, China; ^4^Department of Anesthesiology, Fujian Provincial Hospital, Fuzhou, China

## Abstract

**Objectives:**

This study aimed to evaluate the effect of ultrasound-guided anterior quadratus lumborum block (QLB) at the L2 level in patients undergoing laparoscopic partial nephrectomy.

**Methods:**

Patients who were 18–70 years old with an American Society of Anesthesiologists (ASA) physical status of 1-2 and were scheduled for elective laparoscopic partial nephrectomy were recruited into the cluster randomized controlled trial. Sixty-three patients were randomly allocated to receive QLB (group Q, *n* = 32) or no block (group C, *n* = 31). The patients were not masked to the group allocations. The postoperative follower was blinded to the group allocations. All patients received total intravenous anesthesia, the same multimodal analgesic regimen, and rescue analgesia when needed. The primary outcome was perioperative cumulative sufentanil consumption.

**Results:**

30 patients in group Q and 29 patients in group C were included in the statistical analysis. Block-related complications were not found in this study. Sufentanil consumption during the perioperative period (155.41 [19.58] vs 119.37 [12.41] *μ*g, *p* < 0.001) and sufentanil dosage during surgery and 0–6 h, 6–12 h, and 12–24 h after surgery were lower in group Q than in group C, while 24–48 h after surgery was similar between both groups. The median sensory blockade area in group Q was T9-L1. Comparison of invasive blood pressure (BP) and heart rate (HR) before and after skin incision in group C was statistically significant, but there was no significant difference in group Q. Both at rest and during activity, numerical rating scale (NRS) scores and the incidence of rescue analgesia were lower in group Q at any time point after surgery. The incidences of postoperative nausea and vomiting (PONV), time from postoperative to discharge, postoperative recovery quality, or anesthesia satisfaction were similar between the two groups.

**Conclusions:**

Anterior QLB at the L2 level can reduce the perioperative dosage of sufentanil and the degree of postoperative pain in patients undergoing laparoscopic partial nephrectomy, but it did not improve postoperative recovery quality and anesthesia satisfaction.

## 1. Introduction

Laparoscopic partial nephrectomy is the most common surgical procedure for renal tumors. We wanted to find a method to relieve postnephrectomy pain through research, either by performing surgery laparoscopically or via various anesthesia techniques. Despite the different techniques, chronic postsurgical pain (CPSP) is still a problematic issue which has not been solved yet. Although laparoscopic surgery minimizes the stress response, the postoperative acute pain score and the incidence of CPSP are equivalent to those of open surgery [1], and complete postoperative analgesia can accelerate recovery and prevent the occurrence of CPSP as much as possible [2]. Regional block technology, as a key part of multimodal analgesia, can reduce not only postoperative pain but also the use of opioids after surgery.

Ultrasound-guided quadratus lumborum block (QLB) is a regional block technique in the plane of the abdominal and lumbar fascia with good analgesic effects in various operations. Anterior QLB has a wider range of sensory blocks than other approaches, up to T4-L2 [3], and has a better analgesic effect on visceral pain [4]. The pain after laparoscopic partial nephrectomy is related to the Pfannenstiel incision and deep intra-abdominal pain [5]. Therefore, we consider that anterior QLB can better meet postoperative analgesia needs than other approaches. The initial anterior QLB was described at the L4 level [6], which has also been adopted by most clinical studies [4, 7]. However, a randomized controlled trial (RCT) indicated that anterior QLB at the L2 level produced a widespread cutaneous sensory blockade and a prolonged sensory block compared with the L4 level [8]. At present, there is no clinical trial report on the application of anterior QLB at the L2 level to laparoscopic partial nephrectomy. Thus, this study aimed to evaluate the analgesic effect of ultrasound-guided anterior QLB at the L2 level on perioperative pain management in patients undergoing laparoscopic partial nephrectomy. We hypothesized that ultrasound-guided anterior QLB at the L2 level would provide a significant and clinically relevant reduction in perioperative opioid consumption following laparoscopic partial nephrectomy.

## 2. Materials and Methods

### 2.1. Study Design and Setting

This single-center, randomized controlled study was approved by the Medical Ethics Committee of Fujian Provincial Hospital (K2019-01-001), Fuzhou, China, on January 1st, 2019. This study, which involved human participants, was in compliance with the 1964 Helsinki Declaration and its later amendments. After written informed consent was obtained, 63 patients were enrolled at Fujian Provincial Hospital, China, from January 2019 to December 2019.

### 2.2. Patients

Eligible patients were 18–70 years old with an American Society of Anesthesiologists (ASA) physical status of 1-2 and were scheduled for elective laparoscopic partial nephrectomy. Exclusion criteria were allergy to local anesthetic (LA) and opioids, daily intake of opioids, known abuse of alcohol or medication, local infection at the site of injection or systemic infection, or inability to use a patient-controlled intravenous analgesia (PCIA) pump correctly.

All patients were instructed to use a numerical rating scale (NRS) to describe the degree of pain and a PCIA device (Apollo Science Instrument Co., Ltd., Jiangsu, China) the day before surgery.

### 2.3. Study Interventions

Random numbers were generated by Excel 2016 and placed in sealed opaque envelopes that were consecutively numbered from 1 to 63. Patients were randomly divided into a control group (group C) and a QLB group (group Q) in a 1 : 1 allocation ratio. Approximately 30 min before surgery, patients in group Q were assigned to receive sufentanil 5 *μ*g and then a unilateral ultrasound-guided anterior QLB block at the L2 level. Based on ethics, patients in group C did not receive QLB with isotonic saline. The QLB was operated on by an anesthesiologist experienced in ultrasound-guided nerve block who did not participate in the follow-up data collection. Because of the invasive nature of the interventions, the patients were not masked in the group allocations. The intraoperative anesthesia management and postoperative follow-up were accomplished by the same anesthesiologist who was blinded to the group allocations and followed the patients up to 48 hours postoperatively.

Patients were monitored with a 5-lead electrocardiogram, noninvasive blood pressure (BP), pulse oximetry, invasive radial artery catheterization, and bispectral index (BIS) monitoring (BIS Vista; Medtronic, Minneapolis, MN, USA).

### 2.4. Block Procedure

**Figure 1:**
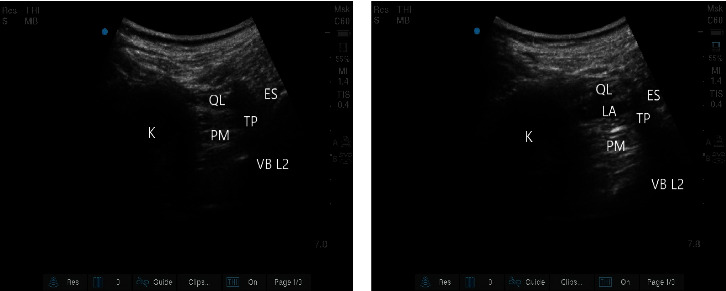
Ultrasonographic images of anterior QLB. (a) Ultrasound anatomical structure before block. (b) Spread of local anesthetic. K, kidney.

Patients were placed in the lateral decubitus position with the surgical side upward. A low-frequency convex probe (SonoSite X-Porte transducer, 2-5 MHz) was placed perpendicular to the spine on the L2 vertebral body (VB), and then the probe was slowly moved ventrally until the L2 transverse process (TP), the quadratus lumborum (QL) muscle, the psoas major (PM) muscle, and the erector spinae (ES) were visualized [8]. A 22-gauge needle (Stimuplex D, B. Braun; Melsungen, Germany) was inserted slowly from the dorsal side to the ventral side using an in-plane technique and passed through the QL muscle before reaching the fascial interspace of the QL and PM muscles. Using a hydro dissection technique, isotonic saline 1*–*2 mL was injected to confirm that the needle tip was positioned correctly, and 25*–*30 mL of 0.5% ropivacaine (NAROPIN; AstraZeneca, London, England) was injected after repeated negative aspiration tests for blood. After injection, the two layers of fascia (fascia of the QL muscle and fascia of the PM muscle) could be seen to separate, and the PM muscle was pressed down, indicating that the drug had spread well (Figure 1). All block procedures were performed in accordance with the principle of sterility. Approximately 30*–*40 min after block completion, a needle was used to test the sensory disappearance plane. The appearance of the sensory loss plane indicated that the block was successful. After blocking, the patients' vital signs were routinely monitored to observe whether there was a local anesthetic systemic toxicity (LAST) reaction.

### 2.5. Surgical Approach

**Figure 2:**
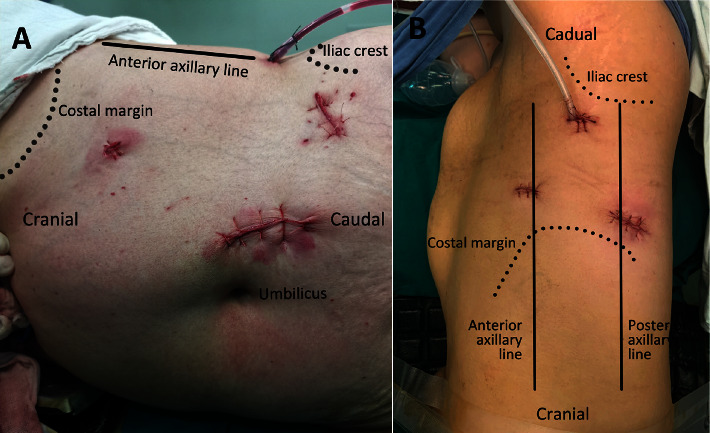
Incisions in the laparoscopic partial nephrectomy. (a) Transperitoneal approach; (b) retroperitoneal approach.

Laparoscopic partial nephrectomy is divided into transperitoneal and retroperitoneal approaches. The patients were positioned in the flank lateral decubitus position. The positions of the trocar for the transperitoneal approach are near the umbilicus (reverse), McBurney's point, under the lateral costal margin of the rectus abdominis, and at the level of the umbilicus of the anterior axillary line. The trocars for the retroperitoneal approach are located 2 cm below the costal arch of the anterior axillary line, 1 cm below the 12th rib of the posterior axillary line, and 2 cm above the anterior superior iliac spine (Figure 2).

The surgical procedures of the two approaches are roughly the same. First, the renal artery and vein were exposed. The renal mass was exposed within Gerota's fascia. A 5 mm margin was marked around the mass with electrocautery. The renal artery was clamped with a laparoscopic bulldog clip. The mass was removed with scissors along the cautery line. Finally, the remaining renal tissue was sutured [9].

### 2.6. Anesthesia and Postoperative Management

All patients received standardized general anesthesia (in order to avoid the analgesic effect of inhalation anesthetics on the study results) with midazolam 2-3 mg, propofol 1–1.5 mg/kg, sufentanil 0.5 *μ*g/kg, cisatracurium besylate, and 0.15 mg/kg for induction were maintained with propofol 3-4 mg/kg/h, remifentanil 0.15 *μ*g/kg/min, and cisatracurium besylate 0.1 mg/kg/h. Anesthetic medications were adjusted according to the BIS value and hemodynamics. Sufentanil 5–10 *μ*g was administered to enhance analgesia if the BP was elevated more than 20% of baseline. If the BP was lower than 20% of baseline, the dosages of propofol and remifentanil were reduced appropriately and fluid therapy and/or norepinephrine infusion was used. During the operation, the BP was maintained within ±20% of baseline (which may be more favorable for patient prognosis improvement), the PetCO_2_ was 35–45 mmHg, and the BIS value was 40–60. Tropisetron 5 mg and flurbiprofen axetil 50 mg were administered intravenously 30 min before the completion of the operation. The PCIA pump was connected immediately after the operation. During the observation period in the recovery room, the patients were again told how to use the PCIA pump when there was moderate to severe pain. The analgesics used in both groups were sufentanil 200 *μ*g and tropisetron 10 mg diluted to a final volume of 200 mL in isotonic saline. The parameters of the PCIA pump included no continuous infusion, 2 mL bolus dose, and 15 min lockout time. When the patients' pain could not be relieved after 2 consecutive bolus doses with the PCIA pump in the ward and the NRS score was still ≥4, flurbiprofen axetil 50 mg was injected for rescue analgesia. If the patients developed nausea and vomiting, tropisetron 5 mg was given intravenously.

### 2.7. Outcomes

The primary outcome was perioperative cumulative sufentanil consumption (*μ*g). Secondary outcomes were (1) invasive BP and heart rate (HR) before and after skin incision; (2) intraoperative dose of propofol (mg), remifentanil (mg), and sufentanil (*μ*g); (3) sufentanil consumption at 0–6 h, 6–12 h, 12–24 h, and 24–48 h after surgery (*μ*g); (4) NRS scores (0–10/10) at rest (supine position) and during activity (defined as changing position from supine to sitting position); (5) nausea and vomiting (yes/no); (6) the incidence of using flurbiprofen axetil after surgery (yes/no); (7) the Quality of Recovery-40 (QoR-40) questionnaire at 24 h after surgery [10]; (8) the Bauer questionnaire at 48 h after surgery [11]; and (9) time from postoperative to discharge (days).

### 2.8. Statistics and Sample Size

Based on a pilot study (10 patients in each group), we expected the total consumption of sufentanil in group Q to be reduced by 30% compared with group C. With *α* set at 0.05 and then for a power of 90% (1 − *β*), we calculated that 27 patients would be needed in each group using PASS software, version 15.0.1 (NCSS LLC., USA). To avoid decreased power as a result of potential dropouts, we enrolled 70 patients and patients from the pilot study were excluded.

Data were analyzed using SPSS software, version 26.0 (IBM Corp., USA). Quantitative variables are summarized as the mean ± SD or median (interquartile range, IQR); qualitative data are expressed as percentage (%) values. Normal distribution was tested using the Shapiro–Wilk test. The quantitative data conforming to a normal distribution were subjected to independent *t* tests or paired *t* tests; otherwise, the Mann–Whitney *U* test or Wilcoxon signed-rank test was used. Qualitative data were compared using the chi-square test or Fisher's exact test. GraphPad Prism software version 9.0.0 (GraphPad Software LLC., USA) was used for graphing. *p* < 0.05 was considered to be statistically significant.

## 3. Results

**Figure 3:**
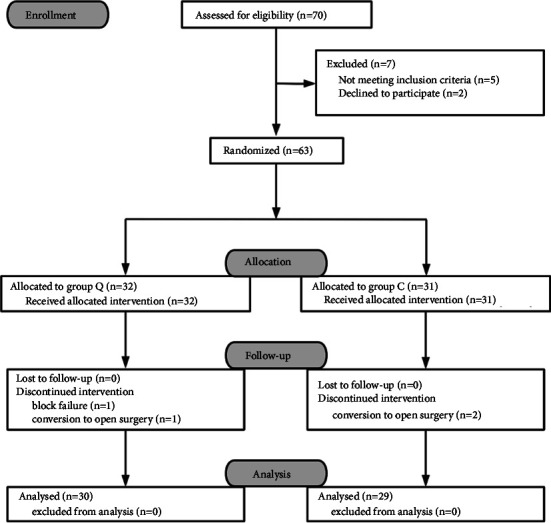
Consolidated standards of reporting trials (CONSORT) diagram.

Among 63 patients who were enrolled in this study, 32 were randomly assigned to group Q and 31 to group C. Eventually, 59 (94%) of the 63 patients completed the trial (Figure 3). Block-related complications such as LAST, bleeding, infection, or neurological deficits were not found in this study. There were no differences in patient characteristics or the dosages of intraoperative propofol and remifentanil between the groups (Table 1).

The total consumption of sufentanil during the perioperative period (*p* < 0.001) and the dosage of sufentanil during surgery and 0–6 h, 6–12 h, and 12–24 h after surgery were lower in group Q than in group C (*p* < 0.001). However, the dosage of sufentanil 24–48 h after surgery showed no significant differences between the two groups (*p* > 0.05; Table 1).

**Figure 4:**
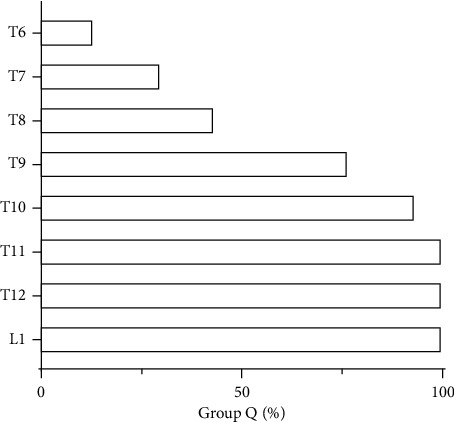
The extent of dermatomal coverage in group Q.

**Figure 5:**
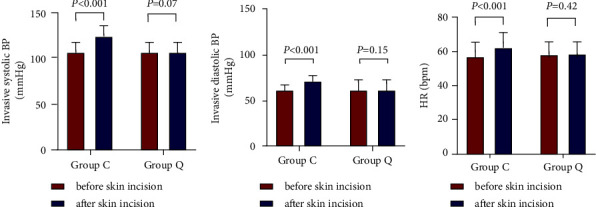
Comparison of BP and HR before and after skin incision.

The median sensory blockade of somatic pain in group Q before anesthesia induction was T9-L1. 4/30 (or 13.33%) of the patients in group Q had the most cephalad dermatomes reaching T6, and the most caudal dermatomes reached L1 in all patients.  Figure 4 shows the extent of dermatomal coverage. Comparison of BP and HR before and after the skin incision in group C was statistically significant (*p* < 0.001), but there was no significant difference in group Q (*p* > 0.05; Figure 5). Both resting and active NRS scores were lower in group Q than in group C at any time point after surgery (*p* < 0.05; Table 2). There was no significant difference in the incidences of postoperative nausea and vomiting (PONV) between the two groups (*p* > 0.05; Table 1). The incidence of rescue analgesia (flurbiprofen axetil) in group Q was lower than that in group C (*p* < 0.05; Table 1). There was no significant difference in time from postoperative to discharge, postoperative recovery quality, or anesthesia satisfaction between the two groups (*p* > 0.05; Table 1).

## 4. Discussion

Common QLB approaches include lateral, posterior, and anterior approaches. Balocco et al. found that the injectates of the lateral and posterior approach were only distributed around the injection point, whereas the injectate of the transverse oblique paramedian (TOP) anterior QLB spread consistently in the anterior aspect of the QL muscle with occasional spread to the lumbar and thoracic paravertebral areas, which indicated that the TOP anterior QLB has a wider block range [12]. A prospective study showed that the application of lateral and posterior QLB to laparoscopic renal surgery did not reduce the consumption of opioids [13], which indirectly verified the observation results of Balocco et al. At present, it is believed that anterior QLB has a wider dermatomal distribution of sensory loss than other approaches of QLB, up to T4-L2 [3, 4], and its diffusion mechanism may be the spread of LA from the thoracolumbar fascia (TLF) to the thoracic paravertebral space, from the posterior to the medial and lateral arcuate ligaments, along the intrathoracic fascia to block the somatic nerves and sympathetic trunk of the lower thoracic segment [14]. QL muscle is encapsulated by the anterior and middle layers of the TLF. There is a high-density network of sympathetic fibers with A and C fiber pain receptors and mechanical receptors on the surface of the TLF, which are sensitive to LA [15]. Therefore, it is generally believed that QLB can not only alleviate somatic pain but also have a certain effect on visceral pain.

At present, research on anterior QLB is mostly performed at the L4 level. However, from the perspective of anatomical structure, below the level of L2, the lateral marginal structures of the paraspinal muscles (TLF, latissimus dorsi muscle, lateral raphe, and the lumbar interfascial triangle) are reinforced by the QL muscle and its fascia; above L2, these structures are only reinforced by the transversalis fascia. Therefore, performing anterior QLB above the level of L2 can make it easier for the LA to spread toward the cranial to the endothoracic fascia in the lower thoracic paravertebral space [16]. Lu et al. found that performing anterior QLB at the L2 level has a wider cutaneous sensory blockade and a prolonged sensory block compared with the L4 level [8]. Based on the anatomical basis and related clinical research, our study chose the anterior QLB at the L2 level. Our results suggest that 30–40 min after block, the pain sensory blockade level was up to T6, and most patients were at T9-L1, which was basically consistent with the above research results.

Patients who undergo laparoscopic partial nephrectomy can develop incision pain, inflammatory pain, and visceral pain due to the surgical incision and the stimulating and stretching effects of abdominal organs and pneumoperitoneum factors [17]. Due to the multiple sources of pain, multimodal analgesia should be adopted for perioperative pain management. As one of the multimodal analgesic approaches, many studies have confirmed that QLB has a good analgesic effect on patients undergoing renal surgery [3, 4, 18, 19]. According to the location of trocars, the plane of somatic analgesia required by the intraperitoneal approach is approximately T8-T12; the retroperitoneal approach is approximately T8-T11; and the side needs to reach the level of the posterior axillary line. In this study, most of the preoperative sensory block plane range was T9-L1. With the passage of time, the spread of this plane may become wider. Therefore, the block plane provided by the anterior QLB at the L2 level can roughly meet the analgesic needs of laparoscopic partial nephrectomy.

Although QLB has a slow onset time and the time to obtain the maximum and stable block range is uncertain [20], all QLB procedures in this study were performed before the operation, and the included cases had a certain range of sensory block planes before the operation. At the same time, it was found that the vital signs of the intervention group did not change significantly before and after the skin incision, and the consumption of sufentanil during the operation could be significantly reduced, indicating that preoperative QLB has an opioid-sparing effect during the operation. The results of this study showed that the sufentanil consumption and NRS score within 24 hours after operation in the intervention group were lower than those in the control group, indicating that the analgesic time provided by ropivacaine for QLB was at least 24 hours, which is consistent with the views of many studies [21]. However, it was also observed that some patients had severe break-out pain 12 hours after the operation, so the duration of analgesia of QLB may vary according to individual differences.

Our results showed that anterior QLB at the L2 level can reduce the perioperative sufentanil dosage and the degree of postoperative pain in patients undergoing laparoscopic partial nephrectomy, but it cannot improve clinical-related events, including the incidence of PONV, the time from postoperative to discharge, the quality of postoperative recovery, and anesthesia satisfaction. The following factors may be involved. First, the female sex is the most important independent risk factor for PONV [22]. In this study, there was no significant sex difference between the two groups, and all patients received tropisetron to prevent PONV before the end of the operation. Second, the influencing factors and quantitative indicators of postoperative recovery quality and anesthesia satisfaction as well as hospitalization time are diverse. The difference between the two groups may affect only pain, with little impact on other aspects. Thereby, the clinical benefits of the opioid-sparing effect and improving postoperative analgesia brought by QLB are limited. However, from the perspective of preventing CPSP, QLB may have certain clinical value. It is considered that poor control of postoperative acute pain and excessive use of opioids are among the factors in the development of CPSP [23]. This study shows that preoperative application of QLB can effectively alleviate pain after laparoscopic partial nephrectomy and reduce the dosage of opioids. In addition, QLB before surgery can reduce or block the nociceptive nerve sensory conduction caused by abdominal incision and reduce the sensitization of peripheral and central pain, which may reduce the risk of CPSP and benefit patients. Therefore, we considered whether QLB can prevent CPSP in patients undergoing laparoscopic partial nephrectomy deserves to be assessed [24, 25].

There are some limitations in our study. First, we were not able to perform a double-blind study due to ethical issues and the patients' awareness of the QLB. Second, due to the coverage of the surgical incision dressing and the lack of clarity regarding the patients' main complaints, the block plane was not measured after the operation. In addition, anterior QLB was applied to the two surgical methods. The analgesic effects may be different, but there was no stratified analysis in this study.

## 5. Conclusions

Ultrasound-guided anterior QLB at the L2 level can reduce the perioperative dosage of sufentanil and the degree of postoperative pain in patients undergoing laparoscopic partial nephrectomy, but it did not significantly improve the quality of postoperative recovery and anesthesia satisfaction.

## Figures and Tables

**Table 1:** Patient's characteristics, primary outcome, and secondary outcomes.

	Group C	Group Q	*P* value
Patient characteristics			
Age, years	53.76 ± 11.37	54.23 ± 12.90	0.88
BMI, kg/m^2^	23.08 ± 2.73	23.45 ± 2.57	0.59
Time of surgery, min	174.24 ± 51.21	179.20 ± 55.42	0.72
Time of anesthesia, min	203.17 ± 49.51	204 ± 54.44	0.96
Sex, female/male	14/15	11/19	0.37
ASA classification, I/II	22/7	20/10	0.44
Approach of surgery, transperitoneal/retroperitoneal	22/7	19/11	0.30
Primary outcome			
The total consumption of sufentanil, *μ*g	155.41 ± 19.58	119.37 ± 12.41	<0.001
Secondary outcomes			
The dosage of intraoperative propofol, mg	768.62 ± 216.91	802.67 ± 240.87	0.57
The dosage of intraoperative remifentanil, *μ*g	1797.24 ± 523.29	1860.33 ± 530.64	0.65
The dosage of intraoperative sufentanil, *μ*g	50.17 ± 9.31	37.17 ± 7.15	<0.001
The dosage of sufentanil 0–6 h after surgery, *μ*g	20 (12, 34)	4 (0, 9)	<0.001
The dosage of sufentanil 6–12 h after surgery, *μ*g	16 (12, 20)	8 (4, 12)	<0.001
The dosage of sufentanil 12–24 h after surgery, *μ*g	36 (26, 36)	26 (24, 28)	<0.001
The dosage of sufentanil 24–48 h after surgery, *μ*g	28 (26, 32)	28 (28, 32)	0.89
Postoperative nausea	7 (24.1%)	4 (13.3%)	0.29
Postoperative vomiting	2 (6.90%)	1 (3.33%)	0.61
Rescue analgesia	25 (86.21%)	16 (53.33%)	0.006
Postoperative recovery quality, score	166.45 ± 8.35	166.53 ± 8.23	0.93
Anesthesia satisfaction, score	42.17 ± 3.95	41.90 ± 4.30	0.80
Time from postoperative to discharge, d	8.24 ± 2.42	8.50 ± 2.73	0.70

Data are either mean (SD), median (IQR), or number of patients (%). BMI, body mass index; ASA, American Society of Anesthesiologists.

**Table 2:** NRS pain scores at rest and during activity in the PACU (T1) and 6 h (T2), 12 h (T3), 24 h (T4), and 48 h (T5) after surgery.

		T1	T2	T3	T4	T5
NRS pain scores at rest	Group C	3.0 (1.5, 3.0)	2.0 (2.0, 3.0)	3.0 (2.5, 3.0)	2.0 (2.0, 3.0)	2.0 (1.0, 2.0)
	Group Q	2.0 (1.0, 2.0)	2.0 (1.0, 2.0)	2.0 (2.0, 3.0)	2.0 (1.0, 2.0)	1.0 (1.0, 1.0)
*P* value		0.004	0.001	<0.001	<0.001	<0.001

NRS pain scores during activity	Group C	4.0 (3.5, 5.0)	4.0 (4.0, 5.0)	5.0 (4.5, 5.0)	4.0 (4.0, 5.0)	3.0 (3.0, 4.0)
	Group Q	4.0 (3.0, 4.0)	4.0 (3.0, 4.0)	4.0 (4.0, 5.0)	3.5 (3.0, 4.0)	3.0 (3.0, 3.0)
*P* value		0.005	0.001	<0.001	0.001	0.004

## Data Availability

The datasets used and/or analyzed for the current study are available from the corresponding authors upon request.
